# Interdisciplinary insights on selection, surveillance and mitigations of antimicrobial resistance dynamics on a UK dairy farm with relevance to other one health sectors

**DOI:** 10.3389/fvets.2026.1829030

**Published:** 2026-08-04

**Authors:** Dov J. Stekel, Ryan Cook, Delveen R. Ibrahim, Michael A. Jones, Diane T. Levine, Chris D. Hudson, Jan-Ulrich Kreft, Jon L. Hobman

**Affiliations:** 1 School of Biosciences, University of Nottingham, Sutton Bonington Campus, Leicestershire, United Kingdom; 2 Quadram Institute Bioscience, Norwich Research Park, Norwich, United Kingdom; 3 Department of Biology, College of Science, The University of Duhok, Duhok, Iraq; 4 School of Veterinary Medicine and Science, University of Nottingham, Sutton Bonington Campus, Leicestershire, United Kingdom; 5 School of Criminology, Sociology and Social Policy, University of Leicester, Leicester, United Kingdom; 6 Centre for Social Development in Africa, University of Johannesburg, Johannesburg, South Africa; 7 School of Biosciences and Institute of Microbiology and Infection, University of Birmingham, Birmingham, United Kingdom

**Keywords:** AMR mitigation strategies, AMR surveillance, antimicrobial resistance, dairy (cows), one health

## Abstract

Antibiotic use in agriculture is a global driver for antimicrobial resistance (AMR). Dairy farming, one of the largest producers of agricultural waste, represents a critical opportunity for mitigating AMR impacts. We review, synthesize and generalize from results of 10 years of interdisciplinary study of AMR in the waste from a single, medium-sized intensive UK dairy farm and related One Health AMR work. Resistance patterns reflect a combination of (i) short-term stability with (ii) long-term change associated with altered antibiotic use. These dynamics were driven by clonal expansion and contraction characterized by chromosomal carriage of resistance, and co-selection by environmentally stable antimicrobials including copper, zinc and tetracycline, rather than horizontal gene transfer. We outline 10 considerations for study, surveillance and mitigation: (i) the need for long-term longitudinal sampling; (ii) the value of intense focus on representative sites; (iii) inclusion of both Gram-positive and Gram-negative sentinels; (iv) adoption of shared standards for measurement and reporting; (v) use of precise language for AMR hazards to enable appropriate study site selection; (vi) reduction of overall antibiotic use, e.g., through infection control; (vii) continued avoidance of veterinary use of internationally recognized critical human antibiotics; (viii) avoidance of environmentally stable antibiotics such as some tetracyclines; (ix) reduction of co-selection pressure via recovery of metals such as copper and zinc; and (x) mitigation of resistance through storage of slurry without further additions, e.g., through the use of two-tank systems. We propose that these considerations are relevant beyond dairy, applicable in wider livestock, environmental and One Health settings.

## Introduction

Antimicrobial resistance (AMR) is a major global challenge, associated with ~1·27 million deaths globally in 2019 (1), predicted to rise to ~1.91 million per year by 2050 (2). Antibiotic use in agriculture leads both to drug-resistant food-borne pathogens (3, 4) and to novel resistance genes transmitting from livestock-associated bacteria into human-acquired infections (5, 6). These can enter humans directly, e.g., through contaminated meat (3, 7), or indirectly, e.g., through manure or slurry applied to soil and entering crops or water (8–10). Many countries have limited the risks of AMR in agriculture (11) by advocating for herd health, hygiene, infection control, prudent antimicrobial usage, and avoiding use of antibiotics for growth promotion and prophylactic treatment [reviewed in (12, 13)]. However, despite the potential for significant reductions, which have been achieved in some sectors, antibiotics must continue to be used to safeguard animal welfare (e.g., 194 tons in British agriculture, UK-VARSS 2025), with consequent risk of selection for and spread of antibiotic resistance genes (ARGs) and antibiotic resistant bacteria (ARB). For countries and sectors that have already made major reductions in antibiotic use, further reductions will be challenging, emphasizing the need for diverse approaches to reduce AMR.

Dairy is a major agricultural industry: the UK is among the 15 largest producers of milk globally, and milk production has the highest monetary value of all UK agricultural outputs. The UK currently has ~7,000 dairy producers holding ~1.62 M cows, with average yield per milking cow of ~8,300 liters per year (14). The UK livestock industry has achieved a 57% reduction in overall antibiotic use between 2014 and 2024, and an 84% reduction in highest priority critically important antibiotics (15). Antibiotics are mainly used for treating mastitis (~23 cases per 100 cows per year) (16) and pneumonia (impacting ~46% of calves) (17), while antimicrobial metals (copper, zinc) or other chemicals (formalin, glutaraldehyde) known to co-select for antibiotic resistance (18, 19) are used to prevent lameness (~31 cases per 100 cows per year) (16). Antibiotics, ARGs and ARBs are mainly released into the environment through waste.

We have been studying antimicrobial resistance for a 10-year period in a dairy farm in the East Midlands of England, typical of well-resourced, high-performance, intensive British dairy farms. We have developed a considerable evidence base that includes longitudinal sampling, knowledge of farm practices, and detailed mathematical models. This perspective article reviews and synthesizes evidence from separate studies, alongside our related work in wider One Health contexts, including comparison with resistance in a nearby major hospital, to understand AMR selection, co-selection and gene transfer. We outline 10 considerations for AMR study, surveillance and mitigations. While these are derived from a single dairy context, in our view they are relevant to livestock production more generally and extend to broader One Health studies.

## The farm

The unit studied was a 220-cow Holstein Friesian herd, with management practices typical for a “high input/high output” UK dairy system. At the time of the study, cows were housed year-round: milking cows housed in cubicles with sawdust bedding; and dry cows on loose straw in yards. Cows were milked through a robotic system and fed a partial mixed ration diet consisting primarily of sileage and concentrate. Annual milk yields were ~10,500 liters per cow.

## Antibiotic use

Antibiotic usage over the study period was typical for similar units at the time. Most doses were intramammary treatments for clinical mastitis (penicillin/aminoglycoside combination therapy); injectable antibiotics in use included oxytetracycline, amoxicillin, cephalexin and benzylpenicillin. Selective dry cow therapy was practiced at the end of lactation, with all cows treated with an internal teat sealant, and infected cows additionally receiving intramammary cloxacillin. Important reductions in antibiotic usage were made between the two sampling periods (2012–14 and 2017–18): fourth-generation cephalosporin (cefquinome) use ceased in August 2015; third-generation cephalosporins (ceftiofur) in January 2016; sulphonamides (sulfadoxine) in June 2016; and first generation cephalosporins (cephalexin) in April 2017.

## Waste handling and flows

Slurry was removed from the cubicle housing by automatic scrapers into a drainage system leading, via a screw press (separating solids from liquids), to a 3,000m^3^ capacity slurry tank. A further 8,000 m^3^ slurry lagoon allowed for additional liquid slurry storage. Soiled bedding from cows housed on straw, alongside solids separated by the screw press, were collected into a separate muck heap. Slurry was used to fertilize arable and pastureland, subject to Nitrate Vulnerable Zone prohibitions between 1 October-31 January.

## Shifting patterns of antimicrobial resistance

**Figure 1:**
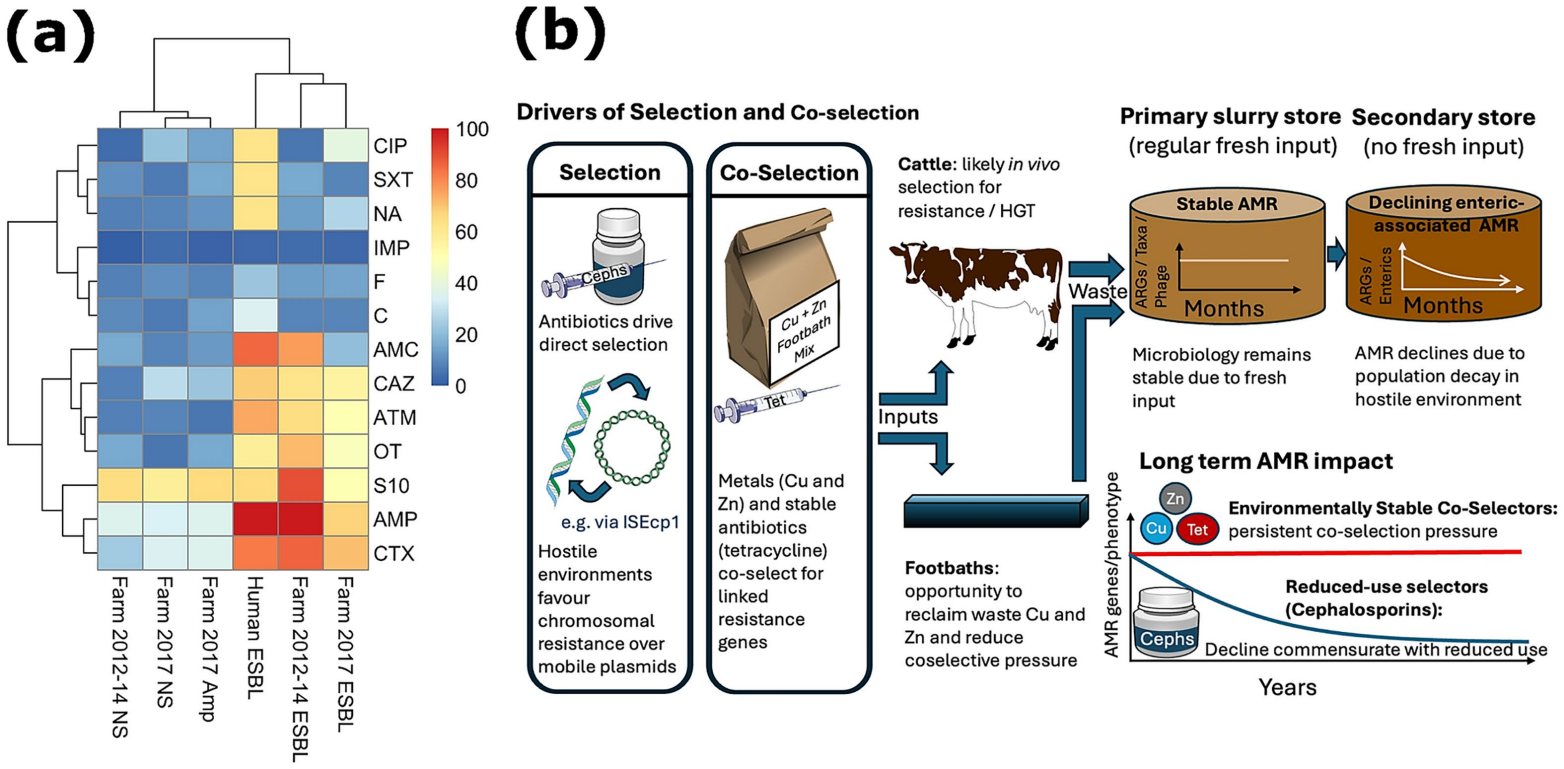
**(a)** Hierarchical clustering of the proportion of isolates phenotypically resistant to the 13 antibiotics tested in common between the two studies on the same farm and a related study of human clinical isolates at a nearby large hospital. (CIP, ciprofloxacin; STX, sulfamethoxazole/trimethoprim; NA, nalidixic acid; IMP, imipenem; F, nitrofurantoin; C, chloramphenicol; AMC, amoxicillin/clavulanic acid; CAZ, ceftazidime; ATM, aztreonam; OT, oxytetracycline; S10, streptomycin; AMP, ampicillin; CTX, cefotaxime). The two main groups cluster by method of isolation (non-selective or Amp versus ESBL). Across these two clusters, there are consistent decreases from the 2012–14 period to the 2017 period in phenotypic resistance in farm samples to almost all beta-lactam antibiotics studied, consistent with cessation of use of cephalosporin and sulphonamide antibiotics in the period between the two studies. The human clinical isolates show higher levels of ciprofloxacin, sulfamethoxazole/trimethoprim, nalidixic acid, nitrofurantoin and amoxicillin/clavulanic acid resistance, consistent with treatment patterns for UTIs and blood infections. Nalidixic acid resistance is strongly correlated with ciprofloxacin resistance; from 2019 it is no longer used in human clinical medicine. **(b)** Schematic of synthesized understanding of drivers for selection and coselection of antimicrobial resistance. Cow: by Aklife via Pixabay, licensed under CC0. Bottle: by OpenClipart-vectors via Pixabay, licensed under CC0. Bag: by Clker-Free-Vector-Images via Pixabay, licensed under CC0. Syringe, Plasmid: provided by Servier Medical Art (https://smart.servier.com), licensed under CC BY 4.0 (https://creativecommons.org/licenses/by/4.0/). DNA: by Vivek Kumar via SciDraw, licensed under CC BY 4.0 (https://creativecommons.org/licenses/by/4.0/).

Antimicrobial resistance profiles of *E. coli* isolates were characterized during two sampling periods: late 2012 to early 2014 (20); and 2017 to early 2018 (21). For One Health comparison, resistance profiles were also characterized in human clinical samples collected in 2016 from patients at the nearby hospital (22). Farm *E. coli* were isolated using non-selective (NS) media (MacConkey or TBX), media supplemented with ampicillin (Amp) or cefotaxime (CTX), or CHROMagar ESBL media (ESBL); the human clinical isolates were all suspected as ESBL-producing *E. coli* on clinical criteria (Figure 1).

**Table 1:** Ten considerations deriving from this work, alongside key advantages and disadvantages of their uptake.

Consideration	Advantages	Disadvantages
(i) Long-term longitudinal sampling	Observe long-term shifts in patterns of resistance	Does not fit well with short-term funding cycles
(ii) Focus on representative sites	Deep knowledge informs interpretation	Fewer sampling locations
(iii) Include both Gram-positive and Gram-negative sentinels	More representative of bacterial pathogens and hosts	Increased costs of culturing and surveillance
(iv) Shared standards for measurement and reporting	Data more comparable between studies	Requires community uptake; reduced flexibility of study design
(v) Precise language to describe AMR hazards	Improved understanding of AMR and study site selection	Requires community uptake
(vi) Reduce overall antibiotic use, e.g., through infection control	Reduced AMR; lower infection rate benefits animals, farmers and other stakeholders	Limit to how far we can go because of need to treat sick animals
(vii) Avoid human critical antibiotics (e.g., 3rd/4th generation cephalosporins)	Reduction in ESCR/ESBL producing bacteria	Potential for increased culling where infections are untreatable (although in practice this appears not to be a major issue)
(viii) Avoid environmentally stable antibiotics, e.g., some tetracyclines	Reduced selection and co-selection for ARGs	Complex stewardship challenge; possible greater reliance on penicillins
(ix) Recovery of metals, e.g., Cu or Zn	Reduced co-selection for ARGs and potential recovery of value	Technology not ready; potential capital investment
(x) Separate storage of slurry, e.g., using two tanks, to allow decline of AMR before field application	Reduced AMR release to the environment	Capital investment costs in two-tank system

While derived from a dairy study, none of these recommendations are dairy-specific and so could have wider relevance to other agricultural systems or environmental contexts.

Comparative clustering of isolates from these three studies reveals an intersection of heterogeneous resistance dynamics (Figure 1a). Isolates primarily clustered by means of isolation, differentiating use of selective media or non-selective media (farm studies) or deliberate choice of putative ESBL-producing bacteria (clinical study). The non-ESBL farm isolates (Farm 2012–14 NS, Farm 2017 NS, Farm 2017 Amp) clustered together and were characterized by generally low resistance frequencies across most antibiotic classes. In contrast, ESBL-positive farm isolates (Farm 2012–14 ESBL and Farm 2017 ESBL) exhibited higher resistance to *β*-lactams (including cephalosporins) together with increased resistance to tetracycline and streptomycin, consistent with selective pressure from veterinary antimicrobial use. The resistance profiles of cephalosporin-resistant strains from the two farm-based studies are more similar to each other than to the human isolates (Human ESBL).

Some of the observed changes in resistance among the farm isolates over the five-year timescale are consistent with changes in farm antibiotic use. Specifically, there were reductions in resistance not only to all cephalosporins, but also to other beta-lactam antibiotics (penicillins and monobactams). The timescale of these changes is consistent with several years of lag between changes in usage and resistance in human AMR in 26 European countries (23).

These long-term changes in resistance in farm isolates contrast with the stability of resistance reported over the 5-month period of 2017 (21). Moreover, the viral community was also stable over this shorter period (24), with 55% of viral operational taxonomic units detected in all samples despite regular emptying and refilling of the tank. The changes in resistance associated with antibiotic use also contrast with consistent presence of MLS resistance genes in the 2017 study, antibiotics that have never been used on the farm. Interestingly, MLS genes are specifically enriched in farm soil amended with this slurry (25), suggesting that MLS resistance represents ecological factors associated with the cattle.

The human clinical isolates showed different patterns of resistance, consistent with typical use of first line antibiotics: amoxicillin for respiratory tract infections, sulfamethoxazole and trimethoprim for urinary tract infections and ciprofloxacin for febrile neutropenia (26). Thus the comparison of these particular data from different One Health settings suggest that resistance patterns are driven by differential antibiotic use in livestock and humans, rather than transmission between sectors. However, the human sample data need to be interpreted with caution as samples may have been cultured for diagnostic purposes. Future One Health assessment of potential transmission pathways could instead focus on regular farm workers with high exposure to farm-associated ARBs and ARGs, or wildlife connected to farms (27).

## Selection, co-selection, and gene transfer

Despite the widely reported importance of HGT in AMR (28–30), we observed high levels of clonality in Extended Spectrum Cephalosporin Resistant (ESCR) *E. coli* across both ERIC-PCR experiments (20) and whole genome sequencing (31). Rather than evidence for HGT, whole genome sequencing found chromosomal *ampC* mutations in 16 of 31 in the first set of sequenced putative ESCR *E. coli* isolates from the farm (32), while further sequencing of 39 cefotaxime resistant *E. coli* strains with similar, but not always identical, antibiotic resistance phenotypes showed chromosomal carriage of IS*Ecp1* elements associated with *bla*_CTX-M-15_ in all strains (31). We also saw clonal contraction in the slurry mesocosm experiments on isolated slurry, with decreases in many genera, including *Escherichia, Klebsiella*, *Pseudomonas, Prevotella* and *Alistipes* (21).

We attribute the observed clonal expansion to co-selection. The IS*Ecp1* elements from the farm showed co-occurrence of ESBL, fluoroquinolone and tetracycline resistance genes (31). Co-selection by the heavily used and environmentally stable oxytetracycline likely drives for their maintenance, especially since fluoroquinolones are not used and cephalosporins are unstable (Figure 1b). While no direct evidence for genetically linked metal and antibiotic resistance genes was found (21), observed Cu and Zn concentrations in the slurry are above minimum co-selection concentrations for those metals (33). Moreover, a farm flow model implicated periodic emptying of footbaths containing Cu and Zn as the only coherent explanation for intermittent spikes of ESCR *E. coli* (32), predicting resistance to be chromosomally carried. This prediction was validated by whole genome sequencing as described above.

Mathematical modelling also explains why the observed AMR dynamics were driven by clonal expansion and contraction rather than horizontal transfer. Models consistently showed that the relative importance of horizontal gene transfer for spread of resistance is highly contingent on bacterial mortality, with HGT occurring only in more benign environments (32, 34, 35). This accounts for both the clonal contraction in the more hostile slurry mesocosm experiments, and the contrasting mobilization of the chromosomal IS*Ecp1* elements into recipient strains under more favorable laboratory conditions with sublethal concentrations of antibiotics (31). Furthermore, the models enable us to hypothesize that the observations from this farm could be extrapolated to other similar environments.

HGT may still occur in this farm environment: there is evidence of carriage of different plasmids in clonal ESCR strains (31). We hypothesize that HGT may occur in the cow gut itself, where temperature and nutrient availability are more favorable to enteric microbiota. This hypothesis is incorporated in other models (36), but we have little direct evidence for this.

## Ten considerations for AMR research, surveillance, and mitigations

We draw out 10 considerations that derive from this and our related work on AMR in agriculture and the environment (Table 1). These include research on slurry-amended soils (25), methods for removal of copper and zinc from agricultural waste (37), standards and language used for the environmental dimensions of AMR (38–40) and broader contributions to interdisciplinary research (41, 42). Agricultural production systems are inherently diverse, and our site represents a specific high-input system. Moreover, dairy cattle are the most long-lived farm animals in many countries; intensive systems with year-round housing necessitate distinct waste handling measures; and farms located in different environments have highly varied natural and human factors. All of these variables have implications for ARB and ARG residence time. Nevertheless, we argue that the following considerations extend beyond dairy and into other agricultural and One Health settings.

### The critical need for longitudinal surveillance over multiple years

Our work showed that this is the timescale on which changes occurred in response to changes of antibiotic use, herd turnover and consequent bacterial population level changes, themselves driven by environmental death rates. This aligns with similar farm-level AMR changes observed in other longitudinal studies in dairy (43). Moreover, working with the same farm for over a decade has facilitated a detailed understanding of its operations, including antibiotic use, slurry and manure storage, herd structure, husbandry practices, and system feedbacks, which permitted sufficiently long sampling to track changes in resistance alongside altered antibiotic use.

### The value of focused, larger interdisciplinary studies on fewer sites

This perspective contrasts with the dominant view that robust evidence must derive from large samples of multiple sites. While such campaigns are valuable, they can miss the uniqueness and relational complexity that characterize many One Health systems. An alternative stance recognizes that some profound insights can only emerge from intensive, multi-method engagement with a single case (44). Such work treats cases as meaningful entities rather than reducible datapoints (45). Judgements about adequacy may draw on concepts of “saturation” or “sufficiency” (46, 47), and increasingly on “information power,” which hold that a smaller number of carefully selected, information-rich cases can generate robust and transferable insights (48, 49). Mathematical modelling can act as a bridge here: mechanistic understanding from well-characterized cases can inform mechanistic models, from which understanding can be generalized, scaled to larger systems and tested against broader datasets.

The single case approach also stems from the value of transdisciplinary engagement with a One Health system (41). The dairy farm collaboration integrated diverse biological, physical and social sciences. Interdisciplinary work is essential for addressing global challenges such as AMR (42), requiring strong communication, shared vision, flattened hierarchies, and supportive funding.

### The importance of monitoring phenotypic resistance of a Gram-positive organism alongside the Gram-negative *E. coli*

*E. coli* is the WHO Tricycle surveillance standard sentinel organism used for monitoring extended spectrum cephalosporin and carbapenem resistance across the world (50), as well as being a WHO critical priority pathogen (51). However, we have seen the importance of Gram-positive associated ARGs, including *tetM* on Tn*916*-like contigs (21), a broad host range gene (52) that persisted in isolated slurry. A potential candidate might be *Enterococcus faecium.* Enterococci are widely used as microbiological water quality indicator organisms (60) and their monitoring is required under the EU bathing water directive (53). Moreover, *E. faecium* is both a WHO critical priority (51) and ESKAPE (54) pathogen. Metagenomics, where feasible, also adds value to surveillance: it was possible to track the metagenome assembled genome of the Gram-negative *Proteiniphilum* from slurry into amended soil, which a focus on easily culturable sentinels would have missed (25).

### The importance of shared standards to enable comparability between studies

The WHO Tricycle protocol (50) provides a clear standard for ESBL-producing and carbapenem resistant *E. coli*. We need similar standards for Gram-positive bacteria. Equally important would be the adoption of a common list of antibiotics to use for AST analysis. The EFSA First Panel contains 14 relevant antibiotics of which 13 (all but colistin) are suited for disk diffusion assays and would form a good basis for harmonized reporting for all *E. coli* (55). We also need shared standards for reporting data and metadata; the Tricycle Protocol and EMBRACE-WATERS standards (38) are relevant examples.

### The use of precise language for AMR hazards when describing field study sites

We have shown that words such as “hotspot,” “reservoir,” and “pristine” do not have coherent meanings in relation to AMR and should be avoided (39, 40). Alternatively, we have suggested that a more appropriate way of describing and justifying study sites is through reporting levels of hazard associated with Prevalence, Transmission, Evolution and Connectivity.

### The need to reduce overall antibiotic use

Overall resistance decreased between the two sampling periods on the farm, commensurate with reduced antibiotic use both locally and nationally. Reduced antibiotic use (e.g., through improved infection control) clearly benefits all stakeholders. For example, the risk of mastitis can be substantially reduced through changes to environmental and other management (56).^1^ This approach may have limitations and often requires infrastructure or ongoing investment to achieve these changes, but this is offset by reductions in disease costs.

### The need for continued avoidance of human critical antibiotics, e.g., 3rd/4th generation cephalosporins

The cessation of 3rd/4th generation cephalosporin use between the two sampling periods led to reductions in resistance to those antibiotics. Similarly, the pattern of resistance in samples derived from the local hospital reflected human antibiotic use. The EMA categorization of antibiotics (57) has been widely adopted within UK livestock agriculture. The UK dairy industry has seen continued progress, with changes to the Red Tractor farm assurance standard restricting use of category B (“restrict”) medicines (including 3rd/4th generation cephalosporins and fluoroquinolones) to very specific/rare circumstances. There has also been a trend towards avoiding category C (“caution”) antibiotics, led by supermarket-aligned and other “higher value” supply chains. However, to be maximally effective, this approach needs international coordination, and political willingness not to import meat or dairy products from countries without these standards.

### The avoidance of environmentally stable antibiotics, such as tetracyclines

Tetracycline resistance persisted in slurry in the mini tank experiments receiving no slurry additions, and IS elements contained tetracycline resistance genes were genetically linked to ESBL-conferring genes. Since many tetracyclines are stable in the environment, it is implicated as a co-selective agent, alongside copper and zinc. However, replacement of tetracyclines with alternatives such as penicillins presents a complex stewardship challenge (58, 59): any substitution should be critically evaluated to ensure that it does not compromise animal welfare, nor inadvertently increase selection for human critical antibiotics.

### The reduction of co-selective pressure by developing methods for recovery of metals such as copper and zinc

Layered double hydroxide adsorbents can remove copper and zinc from spent footbaths (37). This would have considerable benefit in removing key co-selective agents, as well as potentially valorizing what is currently waste material. However, development of affordable and scalable solutions for farms and other industries producing metal pollution is needed.

### The reduction of resistance through storage of isolated slurry before application to land

Storage for 2 months without fresh input was predicted to lead to a 99% reduction of resistance associated with enteric bacteria (21), with 50% reduction in just 4 days. Storage requires filling to alternate between two tanks or other reservoirs, or subdividing an existing tank, so one can be filled with new slurry while the slurry in the other ages before application. Required investment could be incentivized through government grants.

## Concluding remarks

We are of the opinion that these 10 considerations should extend across sectors. Although grounded in dairy research, most are supported by work by ourselves or others in wider contexts. We recognize that the latter three in particular would benefit from further evidence or study. Importantly, we emphasize the value of research that goes beyond description of the phenomenon of AMR, to understand the specific drivers of resistance, thereby informing the most effective surveillance and mitigation strategies within a broader One Health framework.

## Footnotes

1
https://ahdb.org.uk/mastitis-control-plan
